# Novel compound heterozygous mutations in *KLHL24*-induced recessive inherited hypertrophic cardiomyopathy: a case report

**DOI:** 10.3389/fcvm.2026.1771424

**Published:** 2026-05-04

**Authors:** Wenjing Zhou, Xian Wang, Jiyun Yang, Yingnan Liao

**Affiliations:** Genetic Diseases Key Laboratory of Sichuan Province, Department of Medical Genetics, Department of Laboratory Medicine, Sichuan Academy of Medical Sciences & Sichuan Provincial People’s Hospital, School of Medicine, University of Electronic Science and Technology of China, Chengdu, China

**Keywords:** hypertrophic cardiomyopathy, dilated cardiomyopathy, recessive inheritance, Kelch-like family member 24, genetic diagnosis

## Abstract

Hypertrophic cardiomyopathy (HCM) is a common hereditary cardiovascular disease, but the genetic etiology of nearly 50% of cases remains unclear. This case report describes two siblings in a non-consanguineous family who presented with HCM caused by novel compound heterozygous mutations in the *KLHL24* gene. The proband, a 30-year-old male, initially presented with recurrent syncope at the age of 20, leading to the implantation of a permanent pacemaker. Serial clinical evaluations showed progressive left ventricular hypertrophy, diastolic dysfunction, and a gradual decline in left ventricular ejection fraction. His 20-year-old younger brother, identified through family screening, had no obvious clinical symptoms but exhibited severe biventricular hypertrophy, significantly elevated levels of B-type natriuretic peptide and high-sensitivity troponin T, indicating subclinical myocardial injury and heart failure. Genetic testing confirmed that both siblings carried two heterozygous mutations in *KLHL24*: a frameshift mutation (c.532del:p.His178Ilefs*66) in the BACK domain and a missense mutation (c.1514A>G:p.Tyr505Cys) in the KELCH domain. Family verification revealed a recessive inheritance pattern, with each parent carrying one of the mutations. Bioinformatics analysis predicted the pathogenicity of these mutations, which have not been previously reported. This case expands the genetic spectrum of HCM and highlights the importance of genetic testing and family screening for *KLHL24*-related cardiomyopathies. Regular cardiac monitoring is crucial for carriers of such mutations to enable early intervention and improve outcomes.

## Introduction

Hypertrophic cardiomyopathy (HCM) is primarily an autosomal dominant genetic disorder (1, 2), with mutations in *MYBPC3* and *MYH7* accounting for approximately 70% of cases (3–5). However, the genetic basis of nearly half of HCM cases remains undetermined. *KLHL24*, a member of the Kelch-like protein family involved in the ubiquitin-proteasome system (UPS), has recently been linked to cardiomyopathy (6), but related cases are extremely rare. Most previously reported *KLHL24*-related cardiomyopathies are either autosomal recessive cardiomyopathy similar to HCM or autosomal dominant dilated cardiomyopathy (DCM) associated with epidermolysis bullosa simplex (EBS). Notably, there are few reports of recessive inherited HCM caused by compound heterozygous mutations in *KLHL24*, and no such mutations in the BACK and KELCH domains have been documented. This case report describes two siblings with novel compound heterozygous mutations in *KLHL24*, providing new insights into the genetic etiology and phenotypic diversity of HCM. It emphasizes the significance of genetic testing for unexplained HCM and family screening to identify asymptomatic carriers, thereby enriching the scientific literature on *KLHL24*-related cardiovascular diseases.

## Case presentation

The proband (II-1) is a 30-year-old male with no history of hypertension, diabetes, or other chronic diseases. He first presented with recurrent syncope at the age of 20, leading to a diagnosis of HCM and subsequent implantation of a permanent pacemaker. Serial follow-up from 28 to 30 years of age showed progressive disease progression: echocardiography at 28 years revealed asymmetric left ventricular hypertrophy (apical septal thickness 28 mm), left atrial enlargement, and preserved left ventricular ejection fraction (LVEF) of 77%, accompanied by elevated high-sensitivity troponin T (37.70 ng/L) and B-type natriuretic peptide (BNP 815.0 pg/mL). One month later, global left ventricular wall motion incoordination was detected, and LVEF decreased to 70%. At 30 years old, LVEF further declined to 55%, with definitive left ventricular diastolic dysfunction. Global left ventricular wall motion incoordination was noted, along with definitive left ventricular diastolic dysfunction. Right atrial and ventricular leads from the pacemaker were noted to be in a fixed position.

The proband's 20-year-old younger brother (II-2), identified through family screening, had no history of chronic diseases. At 18 years old, echocardiography during family screening revealed extreme asymmetric left ventricular hypertrophy (interventricular septal thickness 38–42 mm). At 20 years old, he reported occasional chest tightness and shortness of breath; further evaluation showed right ventricular hypertrophy with a small right ventricular cavity, LVEF of 56%, significantly impaired left ventricular relaxation (E/e’ ratio 38), and markedly elevated BNP (peaking above 4,600 pg/mL) and high-sensitivity troponin T (60–72 ng/L), indicating subclinical acute heart failure and ongoing myocardial injury.

The proband and his younger brother are from a non-consanguineous family. Their parents (Ⅰ-1, 53-year-old male; Ⅰ-2, 50-year-old female) are clinically asymptomatic. The proband's grandparents had no history of cardiovascular disease.

### Investigations

#### Clinical and imaging investigations

##### Physical examination

The proband (II-1) had a blood pressure of 123/85mmHg, a heart rate of 65 bpm (paced rhythm), a respiratory rate of 20 breaths/min. No precordial elevation was observed, the apical impulse was normally located, and no thrills were palpated. No lower extremity edema or jugular venous distension was noted. His younger brother (II-2) had a blood pressure of 121/58 mmHg, a heart rate of 67bpm, a respiratory rate of 18 breaths/min,. No precordial elevation was observed; the apical impulse was diffuse but without thrills. No lower extremity edema or jugular venous engorgement was present.

##### Electrocardiogram (ECG)

**Figure 1:**
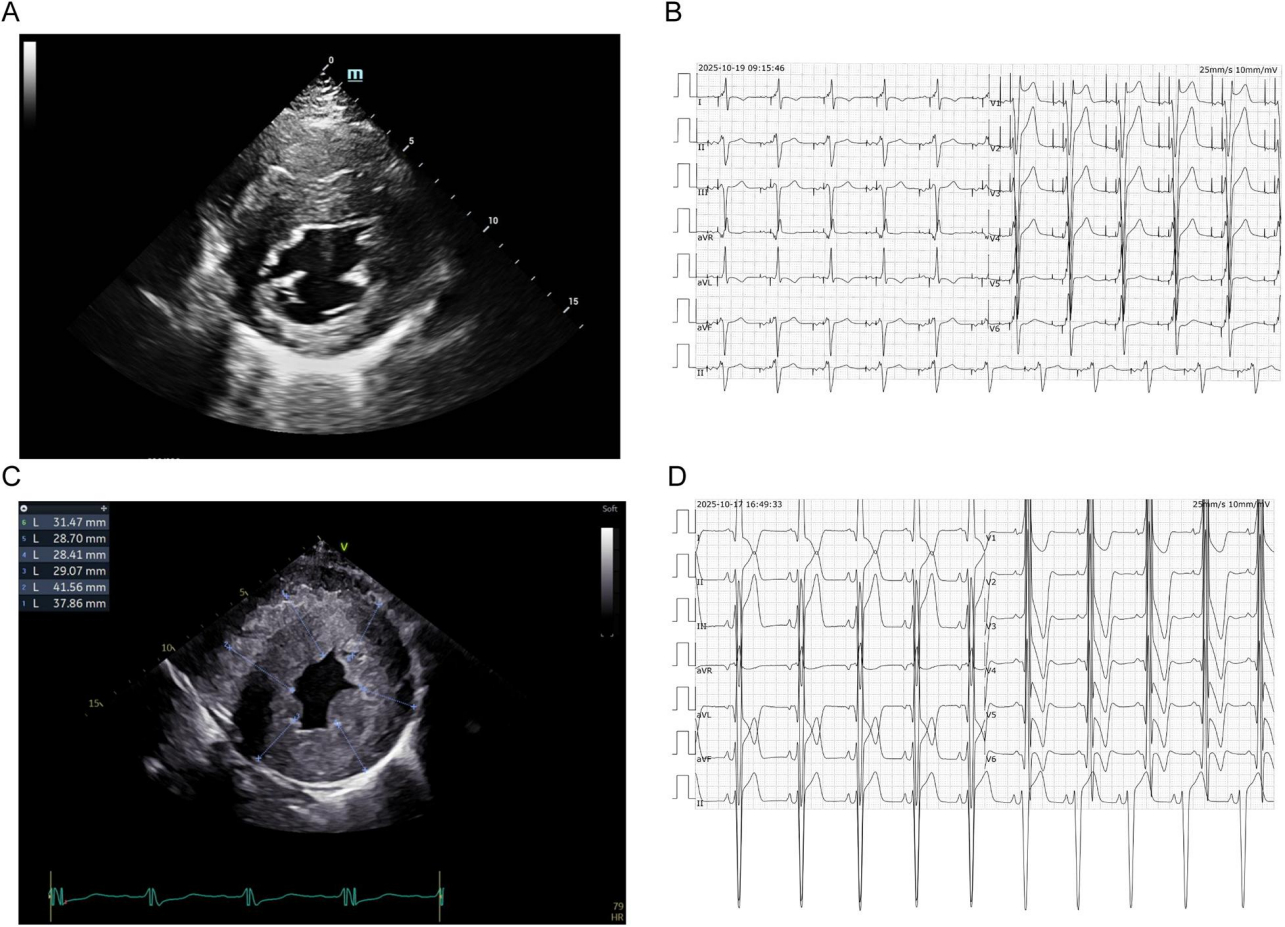
Echocardiograms and 12-lead ECGs of the proband and his brother. **(A, B)** Proband: echocardiogram showing left ventricular hypertrophy, and ECG showing sequential atrioventricular paced rhythm. **(C, D)** Proband's brother: echocardiogram with severe left ventricular hypertrophy, and ECG showing sinus rhythm with left axis deviation (−65°), intraventricular conduction delay, diffuse ST-T changes, and prolonged QTc interval.

The proband had a paced rhythm and atrial-ventricular sequential pacing. The younger brother showed left axis deviation of −65°, intraventricular block, peaked P wave, right ventricular high voltage, left ventricular high voltage, extensive ST-T changes, and prolonged QTc interval (Figures 1B–D). Detailed arrhythmic burden data were unavailable for the proband (II-1) due to his permanent pacemaker. For the younger brother (II-2), 24-hour Holter monitoring (age 18) revealed a mean heart rate of 79 bpm (range 46–136 bpm), 6 single premature atrial contractions (PACs, <0.1%), 7 single monomorphic premature ventricular contractions (PVCs) of two morphologies (<0.1%), and no pauses >2.0 s (longest R-R 1.364 s). No atrial or ventricular tachycardia, couplets, or triplets were observed.

##### Echocardiography

The proband had non-obstructive HCM with asymmetric left ventricular hypertrophy, left ventricular systolic function at the lower limit of normal, reduced diastolic function, and mild tricuspid valve insufficiency. The younger brother had non-obstructive HCM with asymmetric left ventricular hypertrophy, a smaller left ventricular cavity, left ventricular systolic function at the lower limit of normal, diminished diastolic function, right ventricular hypertrophy with a smaller right ventricular cavity, and mild mitral valve insufficiency (Figures 1A–C). Key echocardiographic parameters are shown in Tables 1, 2.

**Table 1:** Clinical characteristics of family members.

Parameter	I-1	I-2	II-1	II-3
Gendar	M	F	M	M
Age, year	53	50	30	20
Age of onset, year			20	
Initial symptoms			Sudden syncope	
ICD years			20	
ECG	Normal	Normal	Left axis deviation of −65°, intraventricular block, peaked P wave, right ventricular high voltage, left ventricular high voltage, extensive ST-T changes, prolonged QTc interval	Paced rhythm, atrial-ventricular sequential pacing
Echo	Normal	Mild tricuspid valve insufficiency	Non-obstructive hypertrophic cardiomyopathy, with asymmetric hypertrophy of the left ventricle; the left ventricular systolic function is at the lower limit of the normal range; the left ventricular diastolic function is reduced. There is mild tricuspid valve insufficiency.	Non - obstructive hypertrophic cardiomyopathy is characterized by asymmetric hypertrophy of the left ventricle, accompanied by a relatively smaller left ventricular cavity. The left ventricular systolic function is at the lower end of the normal range, while the left ventricular diastolic function is diminished. There is also right ventricular hypertrophy with a smaller right ventricular cavity, along with mild mitral valve insufficiency.
LVPW(mm)	5	8	13–14	20–32
IVS(mm)	9	8	20–21	38–42
RVAW(D)(mm)				15
Ejection fraction (%)	69	77	55	56
*KLHL24* mutation	WT/c.532del	WT/c.1514A>G	c.532del/c.1514A>G	c.532del/c.1514A>G

LVPW, left ventricular posterior wall; IVS, interventricular septum; RVAW(D), right ventricular anterior wall in diastole.

**Table 2:** Timeline of the episode of care.

Age	Event	Proband (II-1)	Younger brother (II-2)
18 years	Screening	—	First echocardiography: LVEF 52%, asymptomatic
20 years	Initial symptoms	Recurrent syncope	Occasional chest tightness and dyspnea
20 years	Major intervention	Permanent pacemaker implantation	Initiation of pharmacotherapy (metoprolol, diuretics, etc.)
28 years	First detailed evaluation	LVEF 77%, IVS 28 mm, BNP 815 pg/mL	—
28–30 years	Follow-up	LVEF 70%→55%, diastolic dysfunction developed	—
current	Latest evaluation	LVEF 55%, stable structural abnormalities	LVEF 56%, IVS 38–42 mm, BNP >4600 pg/mL

##### Cardiac MRI

Cardiac MRI data were unavailable for the proband (II-1). In the younger brother, imaging revealed severe left ventricular cavity obliteration, marked interventricular septal thickening (up to 45 mm in systole), and left ventricular outflow tract (LVOT) narrowing with accelerated flow. Late gadolinium enhancement (LGE) demonstrated multifocal, patchy, and linear high signal intensities within the mid-wall of the interventricular septum and its insertion points, confirming extensive myocardial fibrosis and scar formation.

##### Laboratory tests

Both siblings had elevated cardiac biomarkers. The proband's high-sensitivity troponin T and BNP were increased, indicating myocardial microinjury and heart failure. The younger brother had dramatically elevated BNP and persistently high high-sensitivity troponin T, confirming subclinical heart failure and ongoing myocardial injury.

##### Medication history

The proband was on a long-term regimen of metoprolol succinate (47.5 mg daily), spironolactone (10 mg daily), and febuxostat (20 mg daily). His younger brother was discharged on metoprolol succinate (11.875 mg daily), furosemide (20 mg twice daily), tolvaptan (7.5 mg daily), potassium chloride (1 g twice daily), potassium aspartate and magnesium aspartate (two tablets three times daily), and febuxostat (40 mg daily).

#### Genetic investigations

Whole-exome sequencing (WES) was performed using the GenCap liquid-phase capture kit (Maijino, P039-Exome), targeting the entire exome region (hg19). Sequencing was conducted on the Illumina NextSeq 500 platform (paired-end 150 bp reads). Raw data were processed with Cutadapt (adapter removal and low-quality read trimming), BWA (alignment to hg19), Samtools (format conversion) (http://samtools.sourceforge.net/), and GATK (duplicate removal, base quality recalibration) (https://www.broadinstitute.org/gatk/6). Variants were detected using GATK HaplotypeCaller and annotated with Annovar (http://annovar.openbioinformatics.org/en/latest/). Average sequencing depth was 132.46×, with 98.37% of target bases covered at ≥20×. Variant filtering excluded variants with minor allele frequency >2% in public databases (gnomAD, ExAC, 1000 Genomes, ESP6500, and an in-house Chinese control database) and prioritized frameshift, nonsense, splice-site, missense, and in-frame variants. Synonymous and non-coding variants (excluding splice-related regions) were excluded unless previously reported as pathogenic. Candidate variants were retained in genes associated with hypertrophic cardiomyopathy (HCM) and validated by Sanger sequencing in all family members to confirm segregation. Pathogenicity was assessed per ACMG 2015 guidelines (7) using population frequency, functional prediction (REVEL, SIFT, PolyPhen-2, MutationTaster, GERP+), and segregation data. A comprehensive HCM gene panel was screened, including *MYH7, MYBPC3, TNNT2, TNNI3, MYL2, MYL3, TPM1, ACTC1, TNNC1, JPH2, PRKAG2, GLA, LAMP2, RYR2, DSP, FLNC, MYPN, CSRP3, TCAP, ZASP* and others. No pathogenic or likely pathogenic variants in these genes were identified in the proband, his brother, or their parents; only compound heterozygous *KLHL24* variants segregated with the disease phenotype.

Results showed that both the proband and his younger brother carried compound heterozygous mutations in the *KLHL24* gene: a frameshift mutation in exon 3 (NM_017644.3:exon3:c.532del:p.His178Ilefs*66) located in the BACK domain, and a missense mutation in exon 7 (NM_017644.3:exon7:c.1514A>G:p.Tyr505Cys) situated in the KELCH domain. According to the ACMG guidelines, the c.532del mutation was classified as likely pathogenic (PVS1 + PM2_Supporting), and the c.1514A>G mutation was classified as a variant of likely pathogenic with PM3 + PM2_Supporting + PP3_Strong. Bioinformatics tools (REVEL, SIFT, PolyPhen_2, MutationTaster, GERP+) predicted the c.1514A>G mutation to be deleterious (PP3_Strong). These mutations were not reported in the literature, ClinVar database, or general population databases.

**Figure 2:**
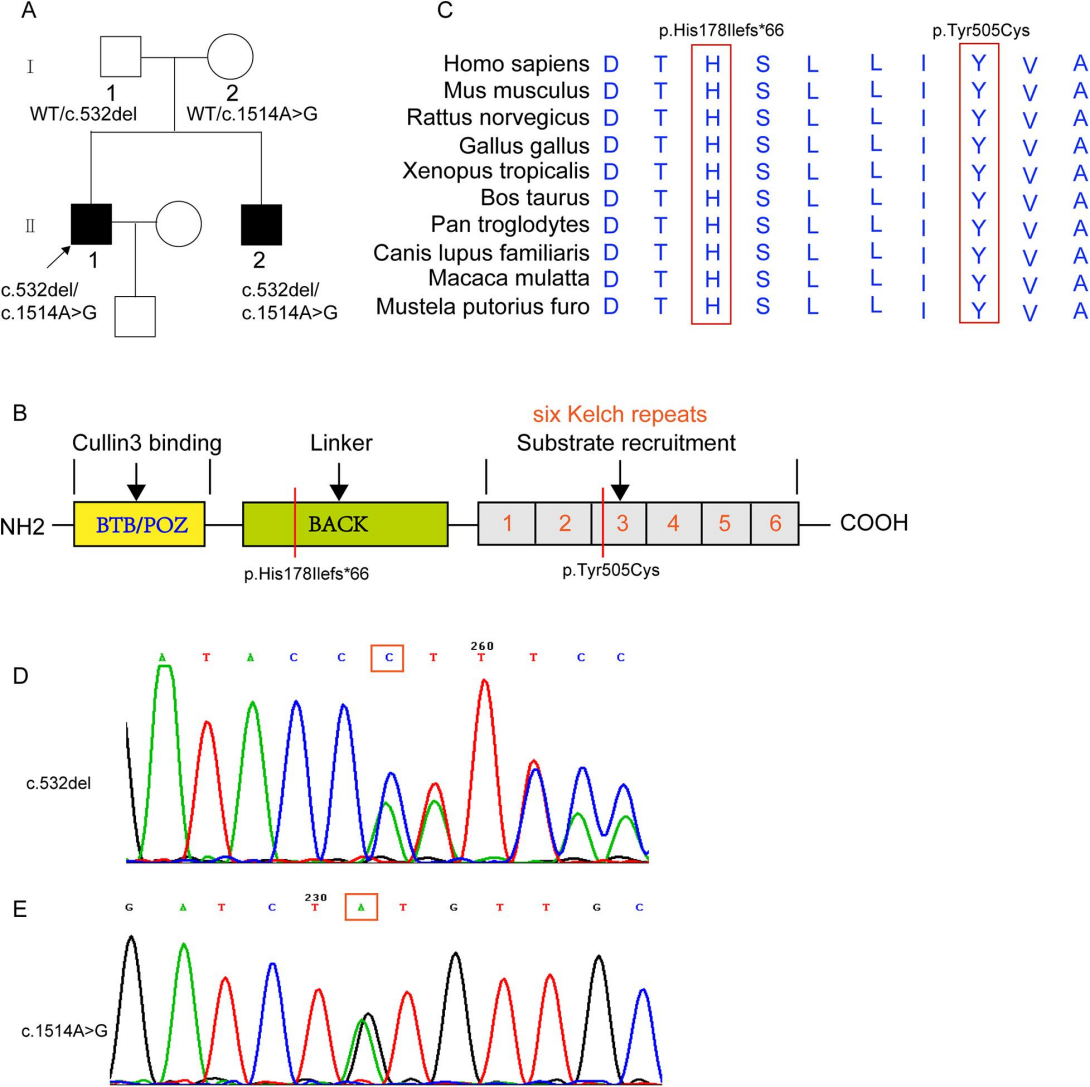
Pedigree chart of a family with *KLHL24* mutations from a non-consanguineous marriage. Solid squares represent individuals with cardiac involvement symptoms. “Wt” indicates the wild - type **(A)**. The schematic diagram presents the domains of *KLHL24* and their corresponding functions. The red vertical lines mark the mutation sites identified in this case **(B)**. The schematic diagram shows the evolutionary conservation of the two mutation sites across ten species. The red boxes mark the amino acid positions where the mutation sites are located **(C)**. The chromatograms show the verification results of the mutation sites c.532del:p.His178Ilefs*66 and c.1514A>G:p.Tyr505Cys by Sanger sequencing **(D,E)**.

Sanger sequencing was performed to verify family members using specific primers (*KLHL24* P472-A4 forward: GAATGTTCGTAATTCAGACATTTCAC; KLHL24 Q472-A4 reverse: CAAGTGATCTATCCACCTCGG; *KLHL24* P472-A5 forward: GAGCTGTGCTCTCAGCCTGT; *KLHL24* Q472-A5 reverse: CAGATCAACGGCACGATAGAC). Results confirmed that the father (Ⅰ-1) carried the c.532del mutation (WT/c.532del), the mother (Ⅰ-2) carried the c.1514A>G mutation (WT/c.1514A>G), and both siblings inherited one mutation from each parent (Figures 2D, E), confirming a recessive inheritance pattern (Figures 2A, B). Conservation analysis revealed that the two mutation sites were conserved across ten species (Figure 2C), suggesting significant impacts on protein structure and function.

Additionally, family members were screened for skin-related symptoms, and no obvious abnormalities were found.

#### Follow-up and outcomes

Both siblings were advised to avoid strenuous exercise, maintain a healthy lifestyle, and undergo regular cardiac monitoring (every 6–12 months) including ECG, echocardiography, and cardiac biomarker detection.

##### Proband

After pacemaker implantation, syncope did not recur. During 10 years of follow-up, his cardiac function gradually declined, but he remained asymptomatic for dyspnea or chest pain and could perform daily activities. At the latest follow-up (30 years old), LVEF was 55%, with stable structural hypertrophy and confirmed left ventricular diastolic dysfunction. He adhered well to the follow-up plan and lifestyle recommendations, with no adverse events related to the pacemaker or disease progression.

##### Younger brother

He had no obvious clinical symptoms but maintained regular follow-up. At 20 years old, echocardiography and laboratory tests showed stable but severe cardiac structural abnormalities and elevated biomarkers. He avoided strenuous exercise and reported good tolerability to the monitoring plan, with no unanticipated adverse events.

##### Patient perspective

The proband (II-1) stated that since receiving his pacemaker, he has had no further syncope and can live and work normally despite gradual decline in heart function. His younger brother (II-2) expressed shock at his diagnosis, as he had never felt unwell, but he adheres to medications and avoids strenuous exercise, hoping for better treatments in the future. The parents (I-1 and Ⅰ-2) stated that although heartbreaking, they are grateful for the diagnosis, will fully cooperate with follow-up, and hope this case can help other families.

## Discussion

This case demonstrates that biallelic, loss-of-function *KLHL24* mutations can cause severe, early-onset recessive HCM, even in the absence of the cutaneous features typically associated with dominant gain-of-function variants. Our report aligns with the severe cardiac phenotype observed in rare cases of homozygous *KLHL24* LOF mutations, yet it is distinguished by its compound heterozygous state and the striking dissociation between profound subclinical disease (in the younger brother) and overt symptoms. Our findings expand the genetic spectrum of recessive HCM and provide mechanistic insights into *KLHL24*-related cardiac dysfunction beyond its known role in keratin regulation.

### Domain-specific functional impact of *KLHL24* variants

*KLHL24* encodes a substrate adaptor of the Cullin3 (CUL3) E3 ubiquitin ligase complex, with two core functional domains: the N-terminal BACK domain and the C-terminal KELCH repeat domain (8, 9).

The c.532del frameshift variant truncates the BACK domain, resulting in complete loss of function (LOF) of the mutant allele. The KELCH domain, by contrast, is responsible for substrate recognition and binding via its *β*-propeller structure. The p.Tyr505Cys missense variant alters a highly conserved residue in the KELCH repeat, disrupting the hydrogen-bond network and substrate-binding pocket, which impairs substrate recruitment and selectivity. In cardiomyocytes, *KLHL24* mediates the ubiquitination and proteasomal degradation of specific target proteins, including desmin—the cardiac homolog of keratin-14 (10). By regulating protein turnover and maintaining proteostasis within the contractile apparatus, *KLHL24* is essential for preserving cardiac structure and function. This dual disruption in our case drives progressive left ventricular hypertrophy, diastolic dysfunction, and myocardial fibrosis—consistent with the siblings’ severe HCM phenotypes.

### Cardiac-specific effects beyond keratin pathways

Prior work focused on KLHL24's role in epidermal keratin regulation (10), but our data and recent functional studies confirm its non-redundant function in the heart. *KLHL24* is highly expressed in cardiomyocytes (11), where it maintains sarcomere turnover, calcium homeostasis, and myocardial structure. Loss of *KLHL24* function leads to: (1) impaired sarcomeric protein quality control, causing myofibril disarray and left ventricular hypertrophy; (2) dysregulated the function of the ubiquitin–proteasome system; (3) accumulation of extracellular matrix proteins, promoting myocardial fibrosis (evidenced by LGE-positive scarring in the younger sibling's cardiac MRI). These effects are independent of keratin pathways, explaining the isolated cardiac phenotype in our patients (no skin abnormalities) and highlighting *KLHL24* as a novel cardiac-specific disease gene.

Research shows that *klhl24a* plays an important role in zebrafish heart development, especially in the formation of a functional ventricle (12). Since hypertrophic cardiomyopathy is essentially a disease characterized by abnormal myocardial structure and function, abnormalities in key genes during heart development may predispose to the occurrence of cardiomyopathy. Therefore, abnormal function of *KLHL24* in heart development may be a potential cause of hypertrophic cardiomyopathy.

### Context with previous *KLHL24* variant studies

Only a small number of studies have linked *KLHL24* to cardiomyopathy: a 2025 study classified *KLHL24* as a moderate-evidence HCM gene (13). Our case aligns with reports of severe, early-onset cardiomyopathy from biallelic *KLHL24* loss-of-function mutations, but is distinct in its compound heterozygous state. Hedberg-Oldfors et al. reported autosomal recessive HCM-like cardiomyopathy in two unrelated families: Family 1 harbored a homozygous nonsense *KLHL24* mutation (c.1048G>T, p.Glu350*) in exon 4 (Kelch domain) within an 8.7-Mb homozygous region; Family 2 had a homozygous missense mutation (c.917G>A, p.Arg306His) in KLHL24, mapped to a 3.4-Mb homozygous region on chromosome 3 in five affected individuals (12). Our patients contrast with the dominant *KLHL24* gain-of-function variants that cause skin fragility followed by DCM (14).

Patients with HCM caused by *KLHL24* variants tend to have an early onset (14). In our case, the proband underwent an implantable device implantation at the age of 20. Although the younger brother has no obvious clinical symptoms at present, at the age of 20, the cardiac ultrasound examination report shows more severe myocardial hypertrophy. Since there are few reported cases of hypertrophic cardiomyopathy type 29 caused by *KLHL24* variants globally, there is currently no epidemiological data on recessive-inherited hypertrophic cardiomyopathy type 29 caused by *KLHL24* variants.

To date, variants such as c.1A > T (p.Met1Leu) (15), c.1A > G (p.Met1Val) (16), c.2T > G (-) (17), c.2T > C (p.Met1Thr) (18), 3G > C (p.Met1Ile), 3G > T (p.Met1Ile) (19), and 3G > A (p.Met1Ile) (18) can cause patients to present with symptoms of generalized epidermolysis bullosa with scarring and alopecia. Among them, the c.1A > G and c.2T > G variants are associated with dilated cardiomyopathy (DCM) (17) and follow an autosomal-dominant, gain-of-function inheritance pattern. Notably, these variants are located at relatively early positions in the gene sequence. Previously reported homozygous loss-of-function (HOM-LOF) variants, c.917G > A (p.Arg306His) and c.1048G > T (p.Glu350*), and the c.532del and c.1514A > G variants detected in our case are located relatively later in the *KLHL24* gene, in the BACK and KELCH domains, respectively.

Several limitations should be acknowledged. First, no functional experiments were performed to validate the functional impact of the identified mutations. Second, follow-up duration was relatively short for the younger brother (only 2 years), limiting our ability to observe long-term outcomes. Third, this study included only a single family with a small sample size, so the generalizability of our findings requires caution.

The primary “take-away” lesson is the imperative for comprehensive genetic screening and aggressive cardiac surveillance in families with HCM. *KLHL24* could be included in genetic panels, especially for cases with early onset, severe hypertrophy, or non-dominant family histories. This case underscores that an “asymptomatic” status in a carrier of biallelic pathogenic variants can be misleading, as severe structural and subclinical functional heart disease may already be present, necessitating pre-symptomatic diagnosis and management.

## Data Availability

The datasets presented in this study are publicly available. The data can be found here: http://doi.org/10.6084/m9.figshare.32064006
